# The Medical Profession: Compulsory or Voluntary Service?

**Published:** 1916-05-20

**Authors:** 


### The Hospital

The Workers' Newspaper of Administrative Medicine and Institutional Life, Administration, National Insurance and Health.

No. 1562, Vol. LX. SATURDAY, MAY 20, 1916. PRICE ONE PENNY.

### THE MEDICAL PROFESSION: COMPULSORY OR VOLUNTARY SERVICE?

Recruiting for the Army has been an anxious national problem for a year or more, and has at last come to a solution in the shape of compulsion. After a wonderful display of volunteered and willing service various expedients were adopted to arouse the more reluctant, but in the end the national situation has necessitated the substitution of selection by law for the privilege of individual choice. At the present moment there is an immediate question whether a similar experience will attend the medical profession. From the date when the need for more doctors was first announced by the Army authorities large numbers of practitioners have voluntarily responded to the call, and have given loyal and effective service to their combatant comrades. The praise and fame of these men are in every land. Now we have come to the second stage, where, by an organised method, an attempt is made forcibly to bring home the necessities of the position to the attention of all members of the profession who are of an age to render military service. Is this to succeed or to fail? The question is an urgent one, and a prompt reply is imperative. If the answer spells failure, there is only one alternative—conscription.

The position is exactly parallel to that provided by the need for fighting men. The authorities have announced in the most solemn manner that there is an immediately urgent need for more doctors for the Army. If these cannot be supplied through the agency of the Central Medical War Committee they must be obtained by other means. And with compulsion in operation on other citizens, public opinion will certainly not shrink from the application of the same method to the medical profession. The time has passed for the consideration of individual interests and individual views. It is the necessities of the nation which are at stake. Equally it is idle to criticise the judgment of the authorities, to say that the doctors already serving are not always wisely distributed, or to jeer at the constitution of the Medical War Committee. Admirable exercises as these might be in times of peace, they are ridiculous or worse at the hour in which we stand. The wants of the situation must be judged by those who carry the responsibility of facing it, and anyone who endeavours to weaken their hands will hardly find either justification or excuse. If this primary position is granted—and voices which contest it have nearly ceased—it inevitably follows that more doctors must promptly give up civil practice and must take service with the Army. Those who have the courage to speak of compulsion are not, as has been said, indulging in a threat. They are stating in plain terms what is a necessary outcome of a possible failure of the scheme to supply on a voluntary basis the medical practitioners which the Army needs.

The situation at the moment can be stated in a few words. The Medical War Committee has asked that all practitioners under forty-five years of age should enrol themselves as willing to accept commissions in the R.A.M.C. should their services be required, the object being both to supply the Army and to secure the medical interests of the civil population. Further, the Committee has announced that it will not feel justified in acting upon these engagements until 75 per cent. of the men concerned have entered their names. All the promises made and received have been asked and given on this undertaking. The reason for such a condition is an obvious one, as it is only in face of what may be called a practically general support that the Committee can accept the responsibility of naming for each man the duty which is incumbent on him.

Only when all are willing can a fair distribution of obligations be arranged. The Committee, of course, is bound by its promise, and thus it cannot call for the redemption of a conditional engagement until the condition named in such engagement is satisfied. Now it is certain that so far the Committee has not proceeded to call upon selected men to join the Army. Equally certain is it that the Army is in urgent want of medical officers. There follows the obvious conclusion that the necessary numbers have not given in their adherence to the scheme of enrolment, and that consequently the active operations of the Committee are for the time being paralysed.

The profession has now to decide—and especially the junior members of it—whether this state of matters is to continue. Is the organised effort to distribute the profession by professional machinery, and in a manner most suitable for the needs of the situation, to break down? Certainly if it breaks down there will be lay intervention. This is made all the more certain by the new Military Service Act, for doctors who have not reached forty-one years of age, equally with their fellow-citizens, must either take service with the Colours or secure exemption by advancing their claims before a local tribunal. The prospect is hardly a pleasing one, either for the individual or for the profession. Yet unless the Medical War Committee is put in a position to carry out its plan this prospect will certainly be realised. There are, we know, some members of the profession, just as there are some members of the public, who favour compulsion rather than free choice on the ground that it affords a nearer approach to equality of sacrifice. It is not our desire either to support or to oppose this view. But we do wish to make the actual situation quite clear and to show that unless the scheme of the Medical War Committee at once commands a general adhesion some rougher method is certain to come into operation.

